# 

**Published:** 1900-06-30

**Authors:** 


### NOTICE TO CORRESPONDENTS.

All MS., Letters, Books for Review, and other matters intended for the Editor should be addressed The Editor, "The Hospital," The Hospital Building, 28 & 29, Southampton Street, Strand, W.C.

The Editor cannot undertake to return rejected MS., even when accompanied by stamped directed envelope.

**Notice to Advertisers and Subscribers.**

All Advertisements, Orders for Copies of The Hospital, and Business Communications should be addressed to **THE MANAGER (Not to the Editor)**, The Hospital, The Hospital Building, 28 & 29, Southampton Street, Strand, London, W.C.

The Cost of Subscription, post free, is as follows:—Per week, 2½d.; per quarter, 2s. 8d.; per half-year, 5s. 3d.; per annum, 10s. 6d. Foreign:—Per annum, 15s. 2d., payable, in all cases, in advance.

**NOTICE.—Vol. XXVII. of "The Hospital" (October, 1899, to April, 1900), in handsome cloth binding, is now on sale at the Office of this Journal, price 7s. 6d. Cases for binding the Numbers contained in this Volume 1s. 6d. Index to Vol. XXVII., 2½d., post free. The Monthly Part for July will be ready on July 2nd. Price 6d., by post 8d.**

### BOOKS RECEIVED.

Kegan Paul, Trench, Trübner, and Co.

"B. Bradshaw's Dictionary of Bathing Places and Climatic Health Resorts."

William Blackwood and Son.

"Things Seen. Impressions of Men, Cities, and Books." By G. W. Steevens. Selected and edited by G. S. Street, with a Memoir by W. H. Henley.

The Scientific Press.

"Burdett's Hospitals and Charities, 1900. Being the Year Book of Philanthropy and the Hospital Annual." By Sir Henry Burdett, K.C.B.

Cassell and Co.

"Diseases of the Tongue." By Henry T. Butlin, F.R.C.S., D.C.L., and Walter G. Spencer, M.S., M.B.Lond., F.R.C.S.

The Athenæum Press, Boston, U.S.A.

"Biological Lectures from the Marine Biological Laboratory of Woods Holl, 1899."

G. Carré et C. Naud, Paris.

"Tonométrie." Par F. M. Raoult.

S. Harvey, Earlestown.

"Annual Report on the Haydock Urban District." By T. E. Hayward, M.B.Lond., Medical Officer of Health.

### Scraps and Gleanings.

The Mercers' Company have made a grant of 50 guineas to Queen Charlotte's Lying-In Hospital, Marylebone, N.W.

The Garrison Fever Hospital, Hull, has been destroyed by fire by order of the Hull Corporation sanitary authority, who have been resolved upon this course ever since the last outbreak of small-pox showed how inadequate and unsuitable the old hospital was.

Mr. J. Passmore Edwards has promised to contribute half the £1,000 required to enlarge the Tilbury Cottage Hospital so soon as the remaining £500 is raised or promised. Already the secretary of the hospital has received £230 towards this £500, so that now there only remains £270 to be provided.

In celebration of the completion of Mr. F. N. Charrington's 50th year, an effort is being made to secure contributions towards clearing off the debt of £20,000 remaining on the Great Assembly Hall, Mile End Road, which he was instrumental in erecting, and towards the cost of which he contributed handsomely.

The receipts of the Victoria Hospital for Nervous Diseases during 1899 were £358 odd, the expenditure being £403 odd. The necessary arrangements for the establishment of a properly equipped intern department are being proceeded with; meanwhile, a ward has been furnished with four beds and all the latest appliances for intern cases.

During 1899 the Leicester Infirmary received from the Hospital Saturday Fund the handsome sum of £3,611, which is an increase of £391 upon the collections from the same source during the previous year. Notwithstanding this increase, the board, owing to a large outlay on account of building and improvements, were in debt to the amount of close on £2,200 at the close of the year.

After a long discussion the minutes of the Bradford City Council, which included a recommendation that the Leeds Road Fever Hospital should be enlarged by the erection of a new wing, were adopted. All were agreed as to the necessity for increased accommodation, but nine out of 50 present were of opinion that an entirely new hospital should be provided on a site removed from the existing buildings.

The Ladies' Committee of the association to provide the troops in South Africa with socks, caps, and other comforts have issued a private report, which is very interesting reading. The work of the committee is now completed, and in the two months during which it was working a very large number of socks, &c., were knitted and dispatched. The Automatic Knitting Machine Company (Limited) were largely instrumental in enabling this result to be achieved, owing to their action in supplying free of cost premises, fitting the same with the necessary number of automatic knitting machines, and providing the services of a competent forewoman and a number of piecework hands, so that ladies could select their wool (which was sold to them at cost price), and then have the articles they wished to send made up without charge by the Automatic Knitting Machine Company.

### BOOKS FOR NURSES.

Domville's Manual for Hospital Nurses, 8th edition ... ... ... ... ... ... ... 2/6

Cullingworth's Monthly Nursing, 4th edition... 1/6

Hellier's Gynæcological Nursing... ... ... 1/6

Cuff's Lectures on Medicine to Nurses .. ... 3/6

Richmond's Antiseptic Principles for Nurses 1/-

Mayne's Short Dictionary of Medical Terms, 2/6

Chavasse's Advice to a Wife, 14th edition, by Dr. Fancourt Barnes ... ... ... ... 2/6

Chavasse's Advice to a Mother, 15th edition, by Dr. George Carpenter .. ... ... ... 2/6

London: J. & A. Churchill, 7, Great Marlborough Street.

### THE NURSE'S REPORT BOOK.

Foolscap 4to., Black cover, 6d. net. By Post 8d.

**Compiled by K. H. (Cert.)**

H. J. Glaisher, 57, Wigmore Street, Cavendish Square, W.

### QUEEN CHARLOTTE'S LYING-IN HOSPITAL

**AND MIDWIFERY TRAINING SCHOOL,**

Marylebone, N.W.

A REPRINT OF

### A NURSING HANDBOOK

TOGETHER WITH S. MAW, SON, AND THOMPSON'S

### COMPLETE CATALOGUE OF NURSING REQUISITES.

The above work is now republished at a cost of 1s. per copy. Messrs. S. M., S., and T. have been compelled to make this charge on account of the extreme popularity of the work and the great expense incurred in its production.

***Post Free on receipt of 1s.***

### S. MAW, SON, and THOMPSON,

7 to 12, ALDERSGATE STREET, LONDON, E.C.

